# Efficacy of Scalp Cooling in Preventing and Recovering From Chemotherapy-Induced Alopecia in Breast Cancer Patients: The HOPE Study

**DOI:** 10.3389/fonc.2019.00733

**Published:** 2019-08-06

**Authors:** Takayuki Kinoshita, Takahiro Nakayama, Eisuke Fukuma, Masafumi Inokuchi, Hiroshi Ishiguro, Etsuyo Ogo, Mari Kikuchi, Hiromitsu Jinno, Naoya Yamazaki, Masakazu Toi

**Affiliations:** ^1^Department of Breast Surgery, National Cancer Center Hospital, Tokyo, Japan; ^2^Department of Breast and Endocrine Surgery, Osaka International Cancer Institute, Osaka, Japan; ^3^Department of Breast Surgery, Breast Center, Kameda Medical Center, Kamogawa, Japan; ^4^Department of Breast and Endocrine Surgery, Kanazawa Medical University Hospital, Kanazawa, Japan; ^5^Department of Medical Oncology, International University of Health and Welfare Hospital, Nasushiobara, Japan; ^6^Department of Radiology, Kurume University School of Medicine, Kurume, Japan; ^7^Department of Diagnostic Imaging, Cancer Institute Hospital of Japanese Foundation for Cancer Research, Tokyo, Japan; ^8^Department of Surgery, Teikyo University School of Medicine, Tokyo, Japan; ^9^Department of Dermatologic Oncology, National Cancer Center Hospital, Tokyo, Japan; ^10^Department of Breast Surgery, Kyoto University, Kyoto, Japan

**Keywords:** scalp cooling, chemotherapy-induced alopecia, breast cancer, hair loss, hair volume recovery, anthracycline, docetaxel, side effects

## Abstract

**Purpose:** This study aimed to assess the efficacy of scalp-cooling devices in preventing chemotherapy-induced alopecia in Japanese breast cancer patients and investigate whether a scalp-cooling device improves hair volume recovery over a 12 weeks period after completing chemotherapy.

**Methods:** This multicenter controlled trial included women with breast cancer undergoing chemotherapy in Japan between February 2016 and March 2018. The primary endpoint was the proportion of patients with no alopecia at the end of chemotherapy. The secondary endpoint included hair volume at 12 weeks after completing chemotherapy.

**Results:** A total of 48 patients were enrolled; of them, 34 and 14 were sequentially allocated to the scalp-cooling group using the Paxman Hair Loss Prevention System and the control group, respectively. There was no significant difference in average age between the scalp-cooling and the control groups (50.0 ± 9.6 vs. 49.0 ± 9.0 years). More than 50% of patients in each group had stage II breast cancer (scalp-cooling group: 53.1%; control group: 64.3%), more than 90% received adjuvant chemotherapy (scalp-cooling group: 96.9%; control group: 92.9%), and more than 60% were treated with a docetaxel/cyclophosphamide regimen (scalp-cooling group: 75.0%; control group: 64.3%). There were more patients judged to have no alopecia at the end of chemotherapy in the scalp-cooling group than in the control group (26.7% [8/30] vs. 0% [0/13]; *P* = 0.011). The proportion of patients with alopecia who experienced an increase in hair volume of ≥50% within 12 weeks duration after chemotherapy was 85.7% (24/28) in the scalp-cooling group and 50.0% (6/12) in the control group. No patient developed serious adverse events related to the scalp-cooling device.

**Conclusions:** The use of a scalp-cooling device prevented alopecia with acceptable safety for Japanese patients. In addition, scalp cooling resulted in faster recovery of hair volume after chemotherapy, even in patients for whom scalp cooling failed to prevent chemotherapy-induced alopecia.

## Introduction

Breast cancer is the commonest cancer in women in Japan, with an incidence of more than 80,000 in 2013 (1). Despite the various adverse reactions, adjuvant chemotherapy is still commonly used for the treatment of breast cancer. Of these reactions, alopecia is among the most distressing for patients (2, 3) and ~8% of patients with breast cancer choose alternative medication to avoid alopecia (4). Although alopecia is not directly life-threatening, it carries a significant psychological burden.

Scalp cooling has been investigated as a method for minimizing alopecia since the 1970s, and advancements in scalp-cooling methods have been made in European countries (5). For example, treatment with a frozen cooling cap has been enhanced by the development of a method to continuously circulate coolant inside the cap so that it no longer needs to be replaced. Two recent prospective clinical studies in the US demonstrated that scalp cooling inhibited alopecia, and two sustained scalp-cooling devices have been approved by the US Food and Drug Administration (6, 7). Scalp cooling is currently the commonest method to reduce chemotherapy-induced alopecia. A meta-analysis of chemotherapy-induced alopecia showed that the use of scalp cooling reduced the risk of alopecia (Relative risk: 0.38, 95% CI: 0.32–0.45), and the rate of hair preservation vary from 10 to 100%, depending on factors, such as chemotherapy regimen, scalp-cooling method, scalp-cooling temperature, and definition of hair preservation (8, 9). According to the latest literature, scalp cooling is more likely to be successful for taxane-based chemotherapy than anthracycline-based chemotherapy (59 vs. 16%) (6).

There are currently two proposed mechanisms for the inhibition of alopecia by scalp cooling. First, cooling constricts blood vessels and reduces blood flow to follicle cells, which may reduce the amount of chemotherapy drug delivered to them. Second, cooling reduces the metabolic activity of follicle cells, which may reduce the cytotoxicity of the chemotherapy agent (10). Meanwhile, it has long been debated whether scalp cooling can preserve cancer cells in cases of micrometastasis prior to cooling (11). However, a recent meta-analysis by Rugo et al. showed that the incidence of scalp metastasis in breast cancer patients is very low, irrespective of cooling, and that cooling does not increase its risk (12).

A recent clinical study based in Japan assessed the efficacy of a scalp-cooling equipment (Paxman Hair Loss Prevention System, UK) and found that scalp cooling reduced the incidence of alopecia after chemotherapy in Japanese patients with breast cancer (13). To build on this finding, the current comparative study aimed to evaluate the safety and efficacy of the Paxman Hair Loss Prevention System in patients with breast cancer. As a secondary endpoint, hair volume was evaluated 12 weeks after chemotherapy termination.

## Materials and Methods

### Study Design and Patients

This multicenter, controlled trial investigated the safety and efficacy of the Paxman Hair Loss Prevention System for preventing or reducing chemotherapy-induced alopecia in breast cancer patients (HOPE; Clinical trial registration no. UMIN000036472). The trial was conducted from February 2016 to March 2018 across five clinical sites in Japan, namely, National Cancer Center Hospital, Osaka International Cancer Institute, Kameda Medical Center, Kanazawa University Hospital, and Kyoto University Hospital. We compared scalp cooling with no treatment. The study included a roll-in group in which patients were trained about wearing a cooling cap, prior to comparison with the scalp cooling and control groups. The main inclusion criteria were female patients with stage I/II primary breast cancer who were scheduled to undergo 4 cycles of adjuvant/neoadjuvant chemotherapy. Target regimens were AC (doxorubicin 60 mg/m^2^ + cyclophosphamide 600 mg/m^2^), EC (epirubicin 100 mg/m^2^ + cyclophosphamide 600 mg/m^2^), TC (docetaxel 75 mg/m^2^ + cyclophosphamide 600 mg/m^2^), or FEC (fluorouracil 500 mg/m^2^ + epirubicin 100 mg/m^2^ + cyclophosphamide 500 mg/m^2^). The main exclusion criteria were alopecia (Common Terminology Criteria for Adverse Events version 4.0 [CTCAE v4.0] Grade 1 or higher) prior to the administration of chemotherapy, history of treatment with chemotherapy, history of migraine, uncontrolled diabetes, thyroid dysfunction, or severe anemia. Reduction of the chemotherapy dose by up to 25% of the defined dose after the second cycle was permitted. Dose delays of up to 2 weeks were allowed.

The study protocol was approved by the Institutional Review Board of all participating institutions, and written informed consent was obtained from all subjects.

### Non-randomization and Intervention

Allocation to each study group was conducted in a sequential, non-randomized manner. At each clinical site, patients were allocated to the scalp-cooling group until the target sample size for the group was reached, after which patients were allocated to the control group. The ratio of allocation to the scalp-cooling group vs. the control group was 2:1 per regimen at each clinical site.

The Paxman Hair Loss Prevention System was used as the scalp-cooling therapy. Scalp cooling was initiated 30 min prior to each chemotherapy infusion, and continued until at least 90 min following completion.

### Outcomes

The primary endpoint was the proportion of patients with no alopecia at the end of chemotherapy treatment. No alopecia was defined as Grade 0 (no hair loss) or Grade 1 (no wig needed, hair loss <50%) based on CTCAE v4.0. Two independent assessors evaluated alopecia based on photographs of the patient's head taken from five directions (front, back, right, left, and above). The highest grade from the five photographs was adopted as the alopecia outcome by each assessor. If both assessors determined alopecia to be ≤ Grade 1, that patient was judged as having no alopecia. The independent assessors received advance training for the evaluation of alopecia and were blinded to group allocation, clinical site, and chemotherapy regimen.

Secondary endpoints included no alopecia (loss of hair volume) assessed by the patient and study doctor; the patients' quality of life (QOL); and hair volume recovery following completion of chemotherapy. Alopecia was assessed by the patient and study doctor at the end of chemotherapy and 12 weeks after the end of chemotherapy using a 5-point scale: 1, no alopecia; 2, 1/2 of hair remaining; 3, 1/3 hair remaining; 4, 1/4 hair remaining; 5, or complete alopecia. QOL was assessed using the European Organization for Research and Treatment of Cancer Quality of Life Questionnaire C30 (EORTC QLQ-C30) and the Hospital Anxiety and Depression Scale (HADS) at baseline, end of chemotherapy, and 12 weeks after the end of chemotherapy. A hair loss-specific questionnaire was also administered at the end of chemotherapy and 12 weeks after the end of chemotherapy. Hair volume recovery following chemotherapy was assessed based on improvement in alopecia grade at 4, 8, and 12 weeks after the end of chemotherapy.

### Statistical Analysis

Data from our previous study (The Dutch Scalp Cooling Registry) (14) were used to calculate the sample size required to achieve 80% power and a 95% confidence interval. The target sample size was determined to be 51 patients; 34 in the scalp-cooling group, and 17 in the control group. The first two patients at each clinical site were considered the roll-in group and were excluded from the analysis sets for efficacy and safety.

The primary analysis set for efficacy was the Per Protocol population, defined as patients who completed all four cycles of chemotherapy among the intention-to-treat (ITT) population (the scalp-cooling group wore the study device at each treatment cycle). The safety analysis set was the ITT population, defined as patients who received at least one cycle of chemotherapy without a major protocol violation among the enrolled patients. Patients with missing data were excluded from the analysis. For the primary endpoint (i.e., the proportion of patients with no alopecia between groups), significance was measured using the Chi square test. All statistical analyses were performed using SAS version 9.4 and JMP version 12.0.1 (SAS Institute, Cary, NC). *P*-values <0.05 were considered statistically significant.

## Results

### Patient Characteristics

**Figure 1:**
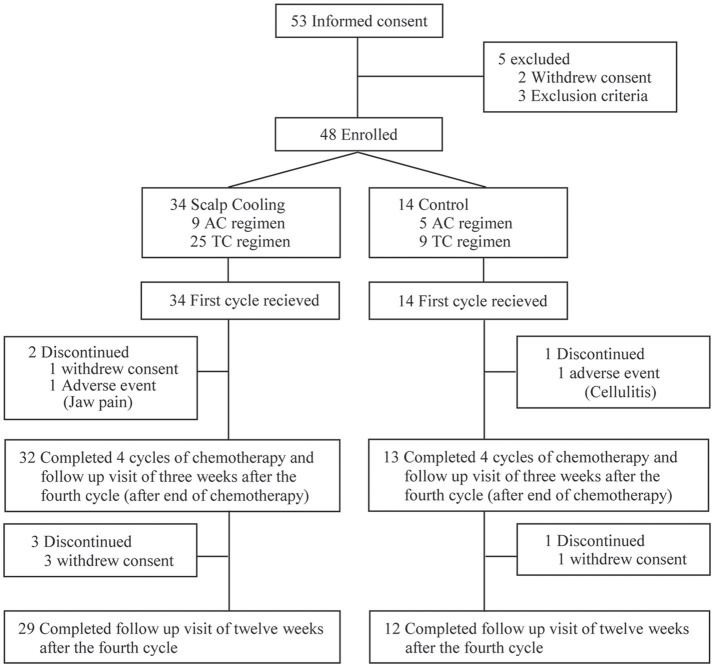
Patient inclusion flowchart.

After the completion of two roll-in patients at each clinical site, 53 patients were recruited for the main part of the study. Of them, five were excluded, and thus 48 were enrolled and allocated to the scalp-cooling group (*n* = 34) or the control group (*n* = 14). All 48 patients started chemotherapy. Two patients in the scalp-cooling group and one patient in the control group were excluded during the follow-up period of the primary endpoint. The remaining 45 patients (32 patients in the scalp-cooling group and 13 in the control group) completed assessment of the primary endpoint 3 weeks after the fourth and final cycle of chemotherapy. Three patients in the scalp-cooling group and one patient in the control group withdrew consent for follow-up observations; thus, they were excluded from the follow-up evaluation (12 weeks after the fourth cycle of chemotherapy). The remaining 29 patients in the scalp-cooling group and 12 in the control group completed the 12 weeks follow-up after the fourth cycle of chemotherapy. Figure 1 details the inclusion and exclusion of patients at each stage of the study.

Two patients with major protocol deviations were excluded in the ITT population, leaving 46 patients: 32 in the scalp-cooling group and 14 in the control group. Both protocol violations were treatment delays due to adverse reactions to chemotherapy, and medical experts considered that an impact on efficacy assessment could not be ruled out. The Per Protocol population comprised 43 patients (30 in the scalp-cooling group and 13 in the control group) after the exclusion of two patients from the scalp-cooling group due to withdrawal of informed consent (*n* = 1 patient) and jaw pain due to overtightened strap (*n* = 1 patient) and of one patient from the control group due to cellulitis.

The patient demographics are shown in Table 1. The average age was similar between the scalp-cooling group and the control group (50.0 ± 9.6 vs. 49.0 ± 9.0 years). More than 50% of patients in each group had stage II breast cancer (scalp-cooling group: 53.1%; control group: 64.3%), more than 90% received adjuvant chemotherapy (scalp-cooling group: 96.9%; control group: 92.9%), and more than 60% were treated with a TC regimen (scalp-cooling group: 75.0%, control group: 64.3%). There were no significant differences in patient demographics between the two groups.

**Table 1:** Patient demographics (ITT population^a^).

		**Scalp-cooling group** ***n*** **=** **32**		**Control group** ***n*** **=** **14**	
Age (years)	Mean ± SD	50.0 ± 9.6	(32)	49.0 ± 9.0	(14)
Breast cancer stage	I	46.9%	(15)	35.7%	(5)
	II	53.1%	(17)	64.3%	(9)
Type of chemotherapy	Neoadjuvant	3.1%	(1)	7.1%	(1)
	Adjuvant	96.9%	(31)	92.9%	(13)
Chemotherapy regimen	AC^b^	25.0%	(8)	35.7%	(5)
	TC^c^	75.0%	(24)	64.3%	(9)

a *The ITT population was defined as patients who received at least one cycle of chemotherapy without a major protocol violation*.

b *Doxorubicin 60 mg/m^2^ + cyclophosphamide 600 mg/m^2^*.

c *Docetaxel 75 mg/m^2^ + cyclophosphamide 600 mg/m^2^*.

### Prevention of Hair Loss

In total, 26.7% (8/30; 95% CI: 14.2–44.4%) of the patients in the scalp-cooling group and 0.0% (0/13; 95% CI: 0.0–22.8%) in the control group were judged to have no alopecia by both independent assessors at the end of chemotherapy. Therefore, the proportion of patients with no alopecia was significantly higher in the scalp-cooling group (*P* = 0.011) (Table 2). Meanwhile, there were 60.0% (18/30) of patients in the scalp-cooling group and 0.0% (0/13) in the control group who were judged to have no alopecia by either one of the independent doctors.

**Table 2:** CTCAE evaluation of alopecia following chemotherapy (PP population^a^).

	**Scalp-cooling group** ***n*** **=** **30**			**Control group *n* = 13**
No alopecia^b^ [95% CI]	8 (26.7%) [14.2%, 44.4%]			0 (0.0%) [0.0%, 22.8%]
Alopecia grade^c^	Grade 0	Grade 1	Grade 2	Grade 2
*n* (%)	1 (3.3%)	7 (23.3%)	22 (73.3%)	13 (100.0%)
Example of head images	^d^ 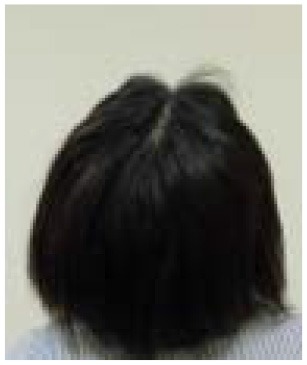	^e^ 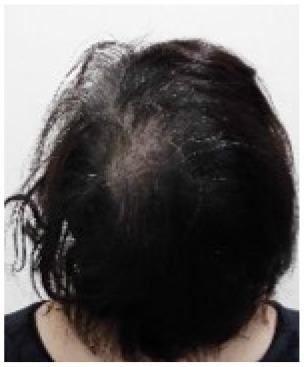	^f^ 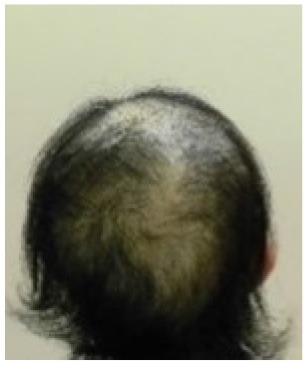	^g^ 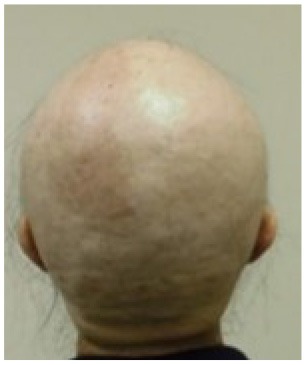

a *The PP population was defined as patients who completed all four cycles of chemotherapy among the intention-to-treat population (the study device was worn at each cycle of chemotherapy)*.

b *No alopecia was defined as Grade 0 or 1 alopecia*.

c *CTCAE v4.0 alopecia grade definition*.
Grade 0 No hair loss Grade 1 Hair loss of <50% of normal for that individual that is not obvious from a distance but only on close inspection; a different hair style may be required to cover the hair loss but it does not require a wig or hair piece to camouflage. Grade 2 Hair loss of ≥50% normal that is readily apparent to others; a wig or hair piece is necessary if the patient desires to completely camouflage the hair loss; associated with psychosocial impact.

d *Parietal view of Grade 0 hair loss at the end of TC chemotherapy, scalp-cooling group*.

e *Parietal view of Grade 1 hair loss at the end of TC chemotherapy, scalp-cooling group*.

f *Parietal view of Grade 2 hair loss at the end of AC chemotherapy, scalp-cooling group*.

g *Parietal view of Grade 2 hair loss at the end of TC chemotherapy, control group*.

### Hair Volume Recovery

The proportion of patients with a hair volume of Grade 0, 1, and 2 at 12 weeks after the end of chemotherapy was 35.7, 50.0, and 14.3%, respectively, in the scalp-cooling group (*n* = 28), and 8.3, 41.7, and 50.0%, respectively, in the control group (*n* = 12).

For patients who were judged as having alopecia at the end of chemotherapy, *post-hoc* analysis was conducted on alopecia recovery. The proportion of patients who recovered from Grade 2 to Grade 0 over the 12 weeks following the end of chemotherapy was 25% (5/20) in the scalp-cooling group and 8.3% (1/12) in the control group. The proportion of patients recovering from Grade 2 alopecia was significantly higher in the scalp-cooling group (*P* < 0.05) (Table 3).

**Table 3:** Recovery from alopecia at 12 weeks after the end of chemotherapy (patients with alopecia at the end of chemotherapy in the PP population^a^).

**Change in alopecia grade at 12 weeks after end of chemotherapy**	**Scalp-cooling group** ***n*** **=** **20**		**Control group** ***n*** **=** **12**		***P*-value^b^**
Grade 2 → 0	25.0%	(5)	8.3%	(1)	0.0393
Grade 2 → 1	60.0%	(12)	41.7%	(5)	
Grade 2 → 2	15.0%	(3)	50.0%	(6)	

a *The PP population was defined as patients who completed all four cycles of chemotherapy among the intention-to-treat population (the study device was worn at each cycle of chemotherapy)*.

b *Wilcoxon ranked sum test (two-sided)*.

### Quality of Life

The analysis of the EORTC QLQ C30 and HADS survey findings revealed no differences between the groups at the end of chemotherapy as well as at 12 weeks after the end of chemotherapy (Table 4).

**Table 4:** Quality of life (ITT population^a^).

	**Baseline (Mean** **±** **SD)**		**End of chemotherapy (Mean** **±** **SD)**			**12 weeks after end of chemotherapy (Mean** **±** **SD)**	
	**Scalp cooling *n* = 32**	**Control *n* = 14**	**Scalp cooling *n* = 30**	**Control *n* = 12**	***P*-value^b^**	**Scalp cooling *n* = 28**	**Control *n* = 12**
**EORTC QLQ-C30 SCORE**							
Global health status	72.66 ± 18.84	69.64 ± 18.38	62.22 ± 23.64	51.39 ± 21.86	0.179	65.77 ± 26.77	65.28 ± 22.14
Emotional functioning	81.25 ± 13.39	77.98 ± 18.95	84.44 ± 13.97	81.25 ± 13.82	0.506	86.90 ± 16.58	84.03 ± 15.27
Social functioning	90.10 ± 15.18	86.90 ± 13.36	77.22 ± 17.77	68.06 ± 21.86	0.165	85.71 ± 14.85	84.72 ± 13.22
**HADS SCORE**							
Anxiety summary	8.88 ± 1.88	9.00 ± 2.57	8.00 ± 2.17	8.42 ± 2.27	0.582	7.89 ± 2.02	8.08 ± 2.02
Depression summary	9.72 ± 1.30	9.50 ± 1.99	9.17 ± 1.90	9.83 ± 1.99	0.316	8.64 ± 1.68	9.17 ± 1.40

a *The ITT population was defined as patients who received at least one cycle of chemotherapy without a major protocol violation*.

b *T-test (two-sided)*.

### Adverse Events

In total, 94% (30/32) of patients in the scalp-cooling group experienced adverse events during the observation period, and 284 adverse events related to the scalp-cooling device were observed. The most common event was jaw pain due to an overtightened strap (75.0% [24/32]). Other adverse events with an incidence of 40% or higher included headache (71.9% [23/32]), discomfort due to chill (68.8% [22/32]), nausea (43.8% [14/32]), forehead pain (40.6% [13/32]), and dizziness (40.6% [13/32]). All cases were successfully managed with appropriate treatment. Serious adverse events were observed in one patient in the scalp-cooling group (fever) and three patients in the control group (acute gastroenteritis, wound infection, skin rash, and cellulitis), but these were unrelated to the scalp-cooling device.

## Discussion

This HOPE study in Japan evaluated the safety and efficacy of a scalp-cooling device in patients with breast cancer who received anthracycline or taxane anticancer therapy. The proportion of patients with no alopecia at the end of chemotherapy was significantly higher in the scalp-cooling group than the control group. In addition, the use of a scalp-cooling device resulted in a significantly higher rate of recovery from alopecia in the 12 weeks following chemotherapy. No safety concerns were identified.

Recent studies showed that scalp-cooling therapy is successful in ~30–80% of patients (Table 5) (6, 7, 14–19). In these studies, the occurrence of alopecia was assessed by the investigators or patients. However, in our study, two independent assessors evaluated the degree of the alopecia using photographs of the patient's head taken from five directions, and the worst assessment was decided as the final outcome. We also believe this is the main factor for the lower success rate (26.7%) of our study compared to other studies. In a previous clinical study on scalp-cooling device in breast cancer patients in Japan, the rate of hair preservation, which was defined as World Health Organization alopecia grade ≤ 2 assessed by clinicians using photographs from one direction, was 52.4% (11/21) in the overall population, 45.5% (5/11) in the anthracycline-based chemotherapy group, and 60% (6/10) in the taxane-based chemotherapy group (13). Using a rather strict definition of successful hair preservation in our study provides strong evidence to support the efficacy of scalp cooling. The racial difference of the head shape may also have influenced our results. The Japanese head shape is more brachycephalic than that of Caucasians (20). Because the cap used for the HOPE study was originally designed for Caucasian head shapes, getting a good cap fit required tightening the cap strap to provide external pressure; this may have resulted in increased hair loss in our patients as proper cap fitting is crucial for successful scalp cooling. This may also account for the higher incidence of jaw pain due to an overtightened strap. A new cap that is better suited to a Japanese head shape has recently been developed, which we hope will be more effective than the current cap.

**Table 5:** Results of recent scalp-cooling studies.

**References**	***N***	**Study design**	**Scalp-cooling method**	**Cancer type**	**Chemotherapy agents**	**Observation period**	**Hair preservation**	**% of hair preservation (controls)**
Nangia et al. (6)	182	Randomized control	Paxman cooling system	Early-stage breast cancer	Taxane and/or anthracycline based	During chemotherapy	CTCAE grade ≦ 1 assessed by independent clinicians	51% (0%)
Smetanay et al. (15)	79	Randomized control	Dignicap cooling system	Breast cancer (stages 1–3)	Taxane and/or anthracycline based	6 months after chemotherapy	Dean grade ≤ 2 assessed by patients	39% (0%)
Rugo et al. (7)	122	Non-randomized controlled	Dignicap cooling system	Early-stage breast cancer	Taxane based	4 weeks after completion of chemotherapy	Dean grade ≤ 2 assessed by patients	66% (0%)
Betticher et al. (16)	238	Non-randomized controlled	Paxman cooling system/Cold Cap	Breast, lung, prostate cancer, etc.	Docetaxel	During chemotherapy	WHO grade ≤ 2 assessed by physician, and no wig required	82% (36%)
Chan et al. (17)	60	No control	Dignicap cooling system	Breast cancer (stages 1–3)	Taxane and/or anthracycline based	2–4 weeks after completion of chemotherapy	Dean grade ≤ 2 assessed by patients	33%
Vasconcelos et al. (18)	131	No control	Paxman cooling system	Breast cancer (stages 1–3)	Taxane and/or anthracycline based	During chemotherapy	Visual evaluation ≤ 50% hair loss assessed by nurse and no wig required	71%
Rice et al. (19)	103	Registry	Penguin Cold Caps	Early or advanced stage breast cancer	Taxane and/or anthracycline based	4 weeks after completion of chemotherapy	VAS ≤ 50% hair loss assessed by patients	61%
Van den Hurk et al. (14)	1411	Registry	Paxman cooling system	Breast, female genital, gastrointestinal/colorectal, lung, prostate cancer, etc.	Various	During chemotherapy	No wig required	50%
Present study	48	Non-randomized controlled	Paxman cooling system	Early-stage breast cancer	Taxane or anthracycline based	3 months after chemotherapy	CTCAE grade ≤ 1 assessed by two independent clinicians	27% (0%)

*VAS, Visual analog scale; WHO, World Health Organization*.

**Figure 2:**
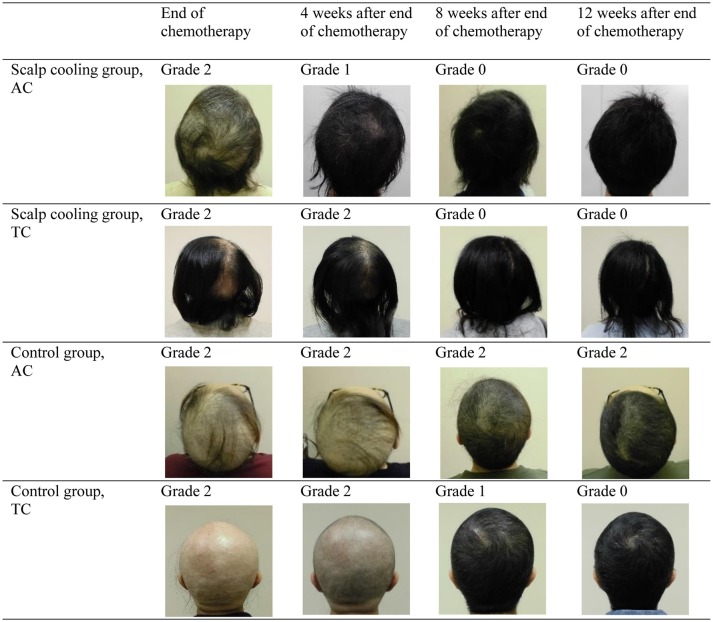
Changes in hair loss following the end of chemotherapy (parietal views; grading by independent assessors).

Chemotherapy-induced alopecia is generally reversible, and hair regrowth is predicted to begin 3–6 months after chemotherapy (21). Although most previous studies of scalp cooling have observed the degree of hair loss during the chemotherapy or 4 weeks after completion of chemotherapy (Table 5) (6, 7, 14–19), Smetanay et al. reported that 6 months after chemotherapy, most patients experienced ≥75% hair recovery regardless of whether or not they used a scalp-cooling device (15). To the best of our knowledge, this is the first study to compare the hair recovery process in detail by independent assessors using a grading scale between patients who did and did not use scalp-cooling treatment. Further, this is the first to show the effect of scalp cooling on hair recovery in chemotherapy-induced alopecia. In this study, 85.0% of patients with Grade 2 alopecia at the end of chemotherapy had experienced an improvement in alopecia 12 weeks after the end of chemotherapy. The degree of recovery was significantly higher in patients using the scalp-cooling device, and the rate of recovery also appeared to be faster in these patients (Figure 2). This study demonstrated an inhibition of and early recovery from alopecia in patients using a scalp-cooling device, which may help to maintain the QOL for these patients. A taxane regimen has been reported to be possibly associated with permanent alopecia or only partial recovery from alopecia after the completion of chemotherapy in some patients (22, 23). However, in our study, recovery from alopecia was better in the scalp-cooling group, despite the higher proportion of patients treated with a taxane regimen in this group (75 vs. 64.3% of the control group). The results from this study suggest that even patients who experience alopecia following chemotherapy benefit from reduced injury to follicle cells by using a scalp-cooling device, consistent with a study by Martin et al. that demonstrated that scalp cooling was effective in the prevention of permanent alopecia (24).

In terms of quality-of-life, there was no difference in EORTC QLQ-C30 and HADS scores between the two groups at 12 weeks after the end of chemotherapy, although the degree of hair recovery was different between the groups. However, both questionnaires used in the present study do not specifically evaluate the impact of hair loss on QOL, and thus they may not be adequately sensitive to detect the impact of alopecia on QOL. A recent systematic review on the effect of scalp cooling on chemotherapy-induced, alopecia-related QOL reported that successful hair preservation using scalp cooling is not consistently associated with QOL outcomes. One reason for this discrepancy is that more than one-third of the studies analyzed QOL in all scalp cooled patients regardless of successful or unsuccessful hair preservation (25). Unfortunately, the sample size in this HOPE study is too small for a subgroup analysis of QOL according to the outcome of the scalp cooling. This is a limitation of our study.

This study evaluated the severity of alopecia based on CTCAE v4.0. However, these criteria consist of only three grades, and we found that patients using the scalp-cooling device had considerable variations in hair volume and length, both between patients and individually, particularly after chemotherapy. This finding may indicate that the scalp-cooling device maintains hair after chemotherapy, resulting in earlier recovery of hair volume. These patients may differ in appearance, and therefore the psychological and social impact may also be different. Accordingly, alopecia assessment using the CTCAE v4.0 might be insufficient for this type of study. In addition to CTCAE v4.0, the severity of alopecia can be objectively assessed using World Health Organization criteria (26) or the Dean Scale (27, 28). However, all of these assessment techniques are based on hair volume and do not include overall appearance, such as hair length and thickness into consideration. In order to determine the value of scalp-cooling therapy in chemotherapy, more valid methods to evaluate the outcome of the scalp cooling should be developed. When developing the new method, the impact on patient satisfaction and QOL should be considered a more important outcome measure as alopecia is not life-threatening.

## Conclusion

Scalp-cooling therapy safely prevents alopecia in patients with an initial onset of stage I/II breast cancer who undergo adjuvant chemotherapy. In addition, scalp cooling results in earlier recovery of hair volume. The development of an alopecia index that considers hair volume, hair length, hair quality, and psychological and social impact is desirable for an appropriate evaluation of the efficacy of scalp-cooling therapy in the future.

## Data Availability

The raw data supporting the conclusions of this manuscript will be made available by the authors, without undue reservation, to any qualified researcher.

## Author Contributions

TK, TN, EF, MI, and HI collected the data. EO, MK, HJ, and NY contributed to clinical analysis. TK and MT wrote the first draft of the manuscript. All authors contributed to the conception and design of the study, contributed to manuscript revision, and approved the submitted version.

### Conflict of Interest Statement

The authors declare that the research was conducted in the absence of any commercial or financial relationships that could be construed as a potential conflict of interest.
